# Real-World Effectiveness, Safety, and Tolerability of Cetosomal Minoxidil 5% Alone and a Fixed Drug Combination of Cetosomal Minoxidil 5% With Finasteride 0.1% in the Management of Androgenetic Alopecia (Inbilt Study)

**DOI:** 10.7759/cureus.41681

**Published:** 2023-07-11

**Authors:** Amit Kerure, Mansukh Ghalla, Satyaprakash Mahajan, Dhiraj Dhoot, Hanmant Barkate

**Affiliations:** 1 Dermatology, Dr Amit Kerure Skin Clinic, Mumbai, IND; 2 Dermatology, Gala Skin and Hair Clinic, Mumbai, IND; 3 Dermatology, Rudranshu Skin Care Clinic, Nashik, IND; 4 Global Medical Affairs, Glenmark Pharmaceuticals Ltd., Mumbai, IND

**Keywords:** india, safety, effectiveness, cetosomal minoxidil, androgenetic alopecia, real world

## Abstract

Introduction

Topical minoxidil 5% is a widely used medication in the treatment of androgenetic alopecia (AGA) but is usually associated with adverse events (AE) such as scalp irritation, dryness, and itching. This prompted the development of nonalcoholic solutions, and cetosomal minoxidil was the most recent one.

Methods

Retrospective multicenter data analysis was conducted at 66 centers across India for adult AGA patients. Patients treated with either cetosomal minoxidil 5% alone (Group I) or a fixed drug combination of cetosomal minoxidil 5% and finasteride 0.1% (Group II) were analyzed for the effectiveness and safety of either formulation. The Physician Global Assessment (PGA) and Patient Global Assessment (PtGA) were used to assess each treatment’s effectiveness. Safety was reported by records of AE and a product tolerability assessment with subjective cosmetic acceptability as recorded by physicians.

Results

Of the 261 patients, 132 were in Group I, and 129 were in Group II. At 16 weeks, in PGA, mild to moderate improvement was noted in 48% and 32% of patients in Groups I and II, respectively, whereas significant to excellent improvement was seen in 52% and 68% of patients in Groups I and II, respectively. Similar results were noted for PtGA. In Group I, 64% of patients rated the product’s tolerability as excellent, and 69% reported the same in Group II. Meanwhile, 64% of patients in Group I and 74% in Group II rated the product as excellent in subjective cosmetic acceptability.

Conclusions

From real-world analysis, cetosomal-based minoxidil solutions were found to be effective and tolerable in AGA and could serve as therapeutic alternatives to alcoholic formulations for AGA management.

## Introduction

Androgenetic alopecia (AGA), commonly known as male pattern baldness, is the most common progressive hair loss disorder, affecting at least 80% of men and 50% of women, with the incidence rising with age [1]. The most commonly affected age range is 30-50 years, with an increasing prevalence of almost 58%. AGA is of great concern because it affects patients’ psychological health and can cause social anxiety and depression [2].

Currently, the US FDA has approved two drugs for the management of AGA: topical minoxidil and oral finasteride [3]. The most commonly used vehicles in topical minoxidil solutions include alcohol, water, and propyl glycol. Most commercially marketed minoxidil solutions face complications such as limited absorption, scalp irritation, dryness, hair frizzing, itching, and inflammation due to their formulations containing ethanol [4]. In the last 3-4 years, nonalcoholic minoxidil solutions have been proven to have a better safety profile than existing solutions [2,5]. Cetosome-based nonalcoholic minoxidil solutions were recently commercialized in India in 2020 as 5% minoxidil (Inbilt® 5%) and a fixed drug combination (FDC) of 5% minoxidil and 0.1% finasteride (Inbilt F®).

Despite this commercialization, there is no clinical data regarding the preparations’ safety or efficacy in real-world settings. Hence, this retrospective data analysis was planned to assess both formulations’ clinical effectiveness and safety in AGA management.

## Materials and methods

**Figure 1:**
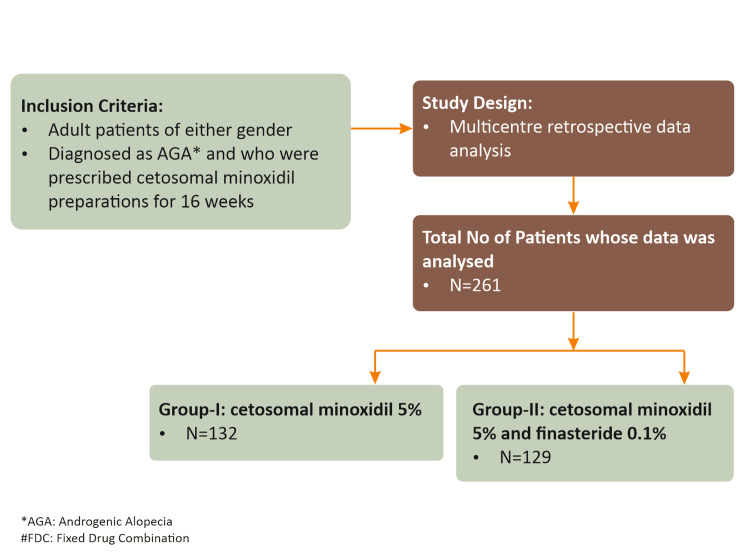
Study design

Retrospective multicenter data analysis was conducted at 66 centers across India. Two hundred and sixty-one patients diagnosed with AGA and treated with either cetosomal minoxidil 5% alone (Group I) or an FDC of cetosomal minoxidil 5% and finasteride 0.1% (Group II) for four months were analyzed for effectiveness and safety of either formulation, as shown in Figure 1. We analyzed effectiveness data on two parameters: the Physician Global Assessment (PGA) and the Patient Global Assessment (PtGA) on a 5-point Likert scale from 0-4 (0: No improvement; 1: Mild improvement [20%]; 2: Moderate improvement [21%-50%]; 3: Significant improvement [51%-75%]; and 4: Excellent improvement [> 75%]). In patients who developed adverse effects (AE), the AE’s type and time to development were recorded. Because this is a nanotechnology-based cetosomal minoxidil formulation, product tolerability assessment and subjective cosmetic acceptability were analyzed on a 4-point Likert scale of 0-3 (0: Poor, 1: Good, 2: Better, 3: Excellent) as recorded by physicians.

This study was approved by an independent ethics committee (Suraksha Ethics Committee, Regd. No. ECR/644/Inst/MH/2014/RR-20). Data analysis was performed using IBM Corp. Released 2011. IBM SPSS Statistics for Windows, Version 20.0. Armonk, NY: IBM Corp. The results were presented as mean scores and compared using paired t-tests with a significance level of 0.05%.

## Results

**Figure 2:**
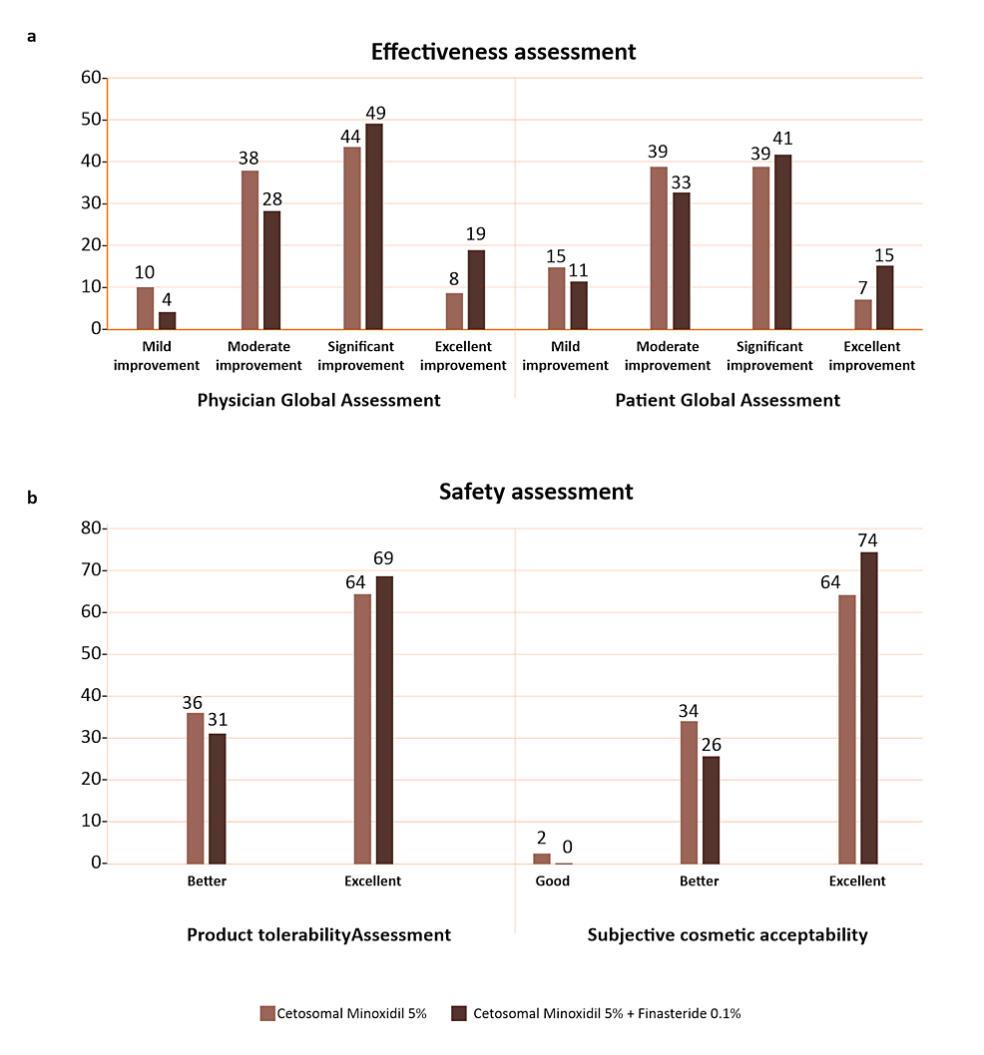
a: Percentage of patients with PGA and PtGA; b: Percentage of patients with product tolerability assessment and subjective cosmetic acceptability

**Figure 3:**
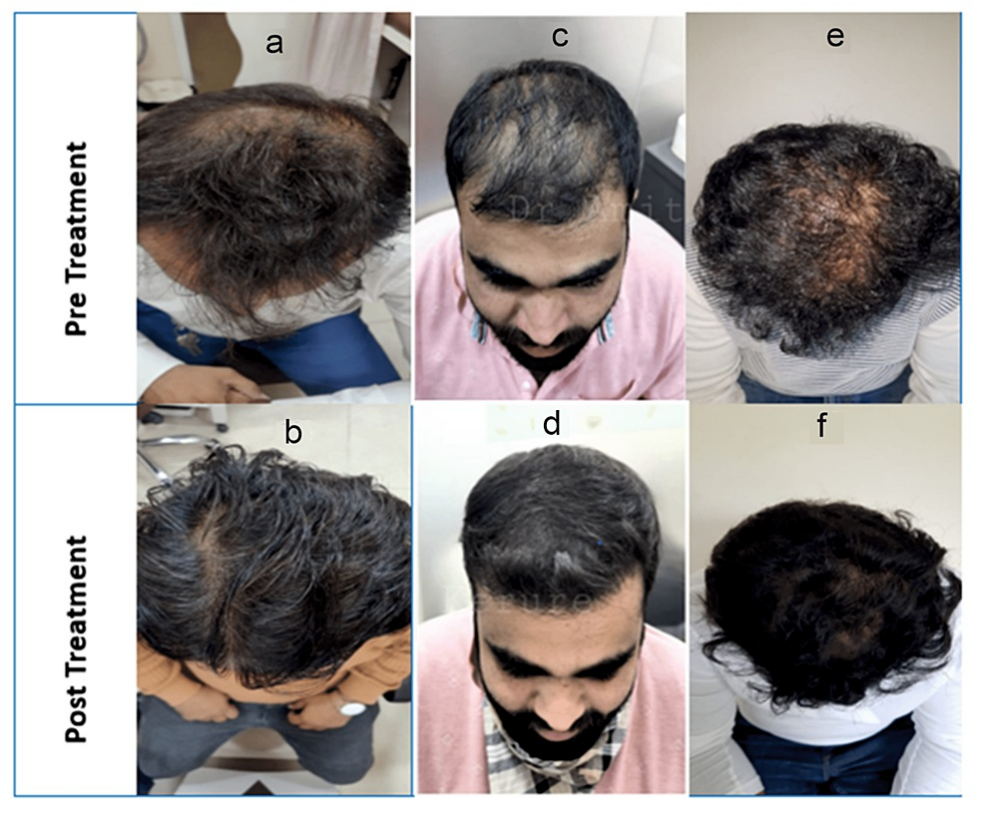
Clinical improvement of AGA before and after treatment; a: before treatment, b: after treatment with cetosomal minoxidil 5% only, (photo courtesy Dr. Mansukh Ghalla) c: before treatment, d: after treatment with cetosomal minoxidil 5% and finasteride combination, (photo courtesy: Dr Amit Kerure), e: before treatment, f: after treatment, cetosomal minoxidil 5% and finasteride 0.1% combination, (photo courtesy: Dr Satyaprakash Mahajan)

Of the 261 analyzed patients, 132 were in Group I and 129 were in Group II. In Group I, the mean age was 34.26 ± 8.83 years, while in Group II, it was 34.30 ± 8.75 years. The mean disease duration was 27.04 ± 29.67 and 21.18 ± 15.20 months in Groups I and II, respectively. At the end of 16 weeks, regarding PGA, 48% of patients in Group I achieved mild to moderate improvement, whereas 52% achieved significant to excellent improvement. In Group II, the same was achieved by 32% and 68% of patients, respectively (Figure 2a). Under PtGA, 54% of patients achieved mild to moderate improvement, whereas 46% achieved significant to excellent improvement in Group I. The same was achieved by 44% and 56% of patients in Group II, respectively. Concerning safety, only one patient in Group I reported scaling, for which treatment was withdrawn temporarily for 10 days and which resolved spontaneously. No patients reported any AEs in Group II. In Group I, 64% of patients rated the product tolerability assessment as excellent, and 69% reported the same in Group II. In Group I, 64% of patients rated the product as excellent in subjective cosmetic acceptability, and it was rated the same by 74% in Group II, as shown in Figure 2b. Clinical improvement in AGA before and after treatment is depicted in Figure 3.

## Discussion

Minoxidil is better absorbed by topical delivery, indicating the need for appropriate delivery formulations. Generally, due to the restrictive nature of the stratum corneum, the drug’s absorption by the topical route depends on the lipophilic properties of the drug, which will influence the amount of skin permeation. Although, in the transappendageal pathway via the hair follicles, the drug avoids the barrier of the stratum corneum, allowing for the diffusion of the drug without interruption [6].

Conventional formulations for topical minoxidil come with various challenges, leading to high levels of drug loss during the application procedure and low patient compliance with the treatment, which may result in unreliable release control of the dosage. To enhance the penetration of the active drug, ethanol or propylene glycol is added to conventional minoxidil formulations. These additions increase the adverse skin side effects in terms of irritation and burning, which may result from the reversion of minoxidil into crystal form upon the solvents’ evaporation. The minoxidil crystals result in the emergence of pruritus, rash, dandruff, and allergic contact dermatitis, which significantly impede patient comfort and cause poor compliance [7]. These AEs led to the development of many non-alcohol-based minoxidil formulations devoid of these cutaneous side effects [2,5,6,8].

In the last 2-3 years, nanoparticles have served as a new drug delivery technology for various physiochemical molecules for different treatments. Nanoparticles offer better drug delivery due to their small size and large surface area, allowing them to be in close contact with the stratum corneum for a prolonged period. The formulation also leads to controlled drug diffusion into the deeper strata of the skin, decreasing the dose needed and loss of the drug with increased efficacy and reduced adverse effects [6].

Among nanoparticles, lipid nanoparticles serve as potential drug delivery alternatives. Cetosomes are part of a novel fast-acting transdermal delivery method related to penetration-enhancing pharmaceutical compositions, comprising a mixture of cetylated esters, cetyl or stearyl alcohols, polar solvents, and surfactants. These combine into amphiphilic nanoparticles within a stabilized liquid dispersion for use in the delivery of medicinal agents through the skin. The compositions in cetosomes enhance topical transdermal fluxes of bioactive without permanently disrupting natural skin barrier function [9].

Among newer drug delivery systems, cetosomes have emerged to increase minoxidil’s stability, provide better skin kinetics, increase therapeutic adherence, and decrease side effects associated with conventional alcohol-based formulations [6].

According to one study, only 26.4% of patients reported having very good product tolerability with conventional topical minoxidil [10]. In our study, > 60% of patients reported excellent product tolerability and cosmetic acceptability. Also, regarding safety, multiple studies on conventional minoxidil have reported AEs, like pruritus (1% to 9%), headache (2% to 4%), and dryness/scaling (2.8%) [11-16]. However, in our study, only 0.3% (1/261) of treatment-related AEs were observed. In terms of efficacy, one study showed that the conventional topical minoxidil solution was found to be very effective in 7.5% of users, moderately effective to effective in 81.3% of users, and ineffective in 6.2% of users [10]. In our study, with cetosomal minoxidil, 8% of patients achieved excellent improvement, and 82% achieved moderate to significant improvement. None of the patients reported ineffective treatment.

Overall, cetosomal minoxidil offers numerous advantages: it avoids the first-pass metabolism, provides a controlled release of minoxidil with a better safety profile, and increases adherence, thus improving the therapy’s overall effectiveness. The retrospective design remains the major limitation, apart from the lack of a comparative arm, yet the results provide helpful information regarding the effectiveness of the cetosomal minoxidil formulation. A comparative clinical study with cetosomal and alcohol-based minoxidil formulations (CTRI/2021/09/036777) is underway to evaluate the efficacy and safety of both formulations in the management of AGA.

## Conclusions

From real-world analysis, novel cetosome-based minoxidil solutions showed a significant improvement in AGA with a favorable safety profile in just four months. This study also demonstrated that the novel minoxidil solution was better tolerated compared with historical data on alcohol-based minoxidil solutions. Hence, these novel cetosome-based minoxidil solutions could be a safer and more satisfactory alternative to conventional alcoholic minoxidil solutions in the management of AGA owing to the chronicity of the disease.
